# Increased Mortality for Elective Surgery during Summer Vacation: A Longitudinal Analysis of Nationwide Data

**DOI:** 10.1371/journal.pone.0137754

**Published:** 2015-09-25

**Authors:** Pascal Caillet, Cécile Payet, Stéphanie Polazzi, Matthew J. Carty, Jean-Christophe Lifante, Antoine Duclos

**Affiliations:** 1 Hospices Civils de Lyon, Pôle Information Médicale Evaluation Recherche, Lyon, F-69003, France; 2 INSERM Research Unit 1033, Lyon, F-69003, France; 3 Université Claude Bernard Lyon 1, Health Services and Performance Research Lab, Lyon, F-69003, France; 4 Hospices Civils de Lyon, Centre Hospitalier Lyon Sud, Service de Chirurgie Générale et Endocrinienne, Pierre Bénite, F-69300, France; 5 Center for Surgery and Public Health, Brigham and Women's Hospital - Harvard Medical School, Boston, Massachusetts, United States of America; Yokohama City University, JAPAN

## Abstract

Surgical safety during vacation periods may be influenced by the interplay of several factors, including workers' leave, hospital activity, climate, and the variety of patient cases. This study aimed to highlight an annually recurring peak of surgical mortality during summer in France and explore its main predictors. We selected all elective of open surgical procedures performed in French hospitals between 2007 and 2012. Surgical mortality variation was analyzed over time in relation to workers leaving on vacation, the volume of procedures performed by hospitals, and temperature changes. We ran a multilevel logistic regression for exploring the determinants of surgical mortality, taking into account the clustering of patients within hospitals and adjusting for patient and hospital characteristics. A total of 609 French hospitals had 8,926,120 discharges related to open elective surgery. During 6 years, we found a recurring mortality peak of 1.15% (95% CI 1.09–1.20) in August compared with 0.81% (0.79–0.82, p<.001) in other months. The incidence of worker vacation was 43.0% (38.9–47.2) in August compared with 7.3% (4.6–10.1, p<.001) in other months. Hospital activity decreased substantially in August (78,126 inpatient stays, 75,298–80,954) in relation to other months (128,142, 125,697–130,586, p<.001). After adjusting for all covariates, we found an "August effect" reflecting a higher risk to patients undergoing operations at this time (OR 1.16, 95% CI 1.12–1.19, p<.001). The main study limitation was the absence of data linkage between surgical staffing and mortality at the hospital level. The observed, recurring mortality peak in August raises questions about how to maintain hospital activity and optimal staffing through better regulation of human activities.

## Introduction

Although more than 230 million major procedures are undertaken worldwide each year [1], the death risk for inpatient surgery remains unexpectedly high [2]. Surgical mortality varies over time within individual hospitals, suggesting that there is opportunity for improvement. Part of this variability is from occasional safety issues, provoking a momentary mortality peak in particular institutions [3]. Another component may relate to the common patterns of seasonality across institutions, as reflected through the recurring death peaks in many hospitals during the same period every year. For example, there is a well-known annual July effect in teaching hospitals at the beginning of a new academic year. Increased surgical mortality is correlated to the influx of inexperienced trainees and the lack of attending oversight, which is possibly related to vacation schedules [4]. Furthermore, there is a higher risk of death for patients who undergo elective surgical procedures later in the working week and on the weekend [5,6].

Among the members of the Organization for Economic Cooperation and Development [7], France is the most generous country with respect to the legal right to paid leave [8]. Workers have six weeks of paid vacation annually on average, and it is common for them to take their longest annual holiday in summer [9]. Due to this recurring annual phenomenon, there may be a risk for patients undergoing surgery in institutions with reduced capacity. Vacation periods can place surgical teams under great pressure and alter the quality of surgery [10]. Inadequate staffing [11] and the inability to maintain sufficient procedural volumes may transiently jeopardise surgical outcomes [12]. High ambient temperatures may also lead to increased mortality [13] as well as cluster patients with poor prognoses and risky comorbidities whose care cannot be delayed.

We hypothesised there might be an “August effect” in France that is characterised by an annually recurring peak in surgical mortality. Using nationwide databases, we aimed to determine its main predictors among vacation periods, hospital activity, climate factors, and the patient case mix.

## Materials and Methods

### Data

We designed a simultaneous longitudinal analysis of the following three data sources: the nationwide hospital database in acute care, the continuous employment survey among workers, and the national weather newsletter. All elective open surgical procedures performed in French hospitals were selected. The mortality variation was interpreted over time in relation to workers leaving on holiday, the volume of procedures performed within hospitals, and temperature changes.

The nationwide hospital database was obtained for patients discharged over a 6-year period, from January 2007 through December 2012. This database includes information about all inpatient stays in every public and private hospital with acceptable validity [14]. For example, in 2010, the French population totalled 62,765,235 persons [15], resulting in 16,709,216 acute hospitalisations [16]. The standard discharge abstracts for each of these hospitalisations contain compulsory information about the patient (e.g., gender and age) and the patient’s primary and secondary diagnoses, using the International Classification of Diseases, 10^th^ revision (ICD-10 codes), as well as procedural codes associated with the care provided.

Data about the workers’ vacations were obtained from the National Institute of Statistics and Economic Studies in the form of a continuous employment survey aimed at observing the situation of the French work market as part of the European Union labour force survey. Starting in 2001, this survey has continuously logged the work status of 8,000 new people every week, aged from 15 to 75 years old [9].

Climate data were provided by the national weather service. These data are gathered from a network of meteorological stations that are scattered all over the French territory. To monitor seasonal temperature variations, the mean of the maximum daily temperatures was calculated monthly in a hundred geographical areas [17]. Depending on its location, every hospital was then linked to a single geographic area with the corresponding set of temperatures over the study period.

### Population

From the French hospital database, all adults who were 18 years of age or older and who underwent open surgery and possibly died during their hospitalisation were identified. To preserve the homogeneity of the sample as well as exclude unavoidable deaths, ophthalmologic, dental, and obstetric procedures were not selected; we also excluded paediatric, ambulatory and palliative care, and organ retrieval cases. Because outpatient surgery is associated with a very low rate of mortality, we further restricted our analysis to inpatient stays with at least one night spent in the hospital. According to the above criteria, 12,793,516 stays with surgical procedures performed in 1,330 hospitals were extracted. We then identified elective procedures that reflected all surgical specialties to enhance the generalisability of the results across the spectrum of surgical care and hospitals. We chose to exclude high-risk emergency cases from the dataset because the corresponding flow of inpatient admissions could not be properly regulated. Finally, given our interest in longitudinally monitoring surgical mortality, we further limited the study population to hospitals that provided continuous care over 72 months, where at least one death was recorded per year, and with at least 100 surgical procedures per year, over the six-year period. Therefore, we were expecting to minimise artefacts from systematic coding errors or nonadjustable case-mix variations. With these exclusions, our final study cohort consisted of 8,926,120 inpatient stays in 609 facilities.

### Variables

Our primary outcome measure was in-hospital death within 30 days of patient admission. To adjust mortality for case-mix variations, our set of covariates included the patients’ age, gender, and 31 coexisting conditions extracted from the Elixhauser list of comorbidities [18]. For every procedure, the anatomical site of the surgery was also considered and complexity index was controlled for based on the level of human and material resources involved in performing the procedure.

Additionally, to account for structural or environmental determinants, several variables were considered at the hospital level, including the facility type (public, private, or teaching), volume of surgical procedures performed monthly, and geographic location (North West, North East, South West, South East, or Paris). To measure the potential activity reduction in August at every hospital, we first calculated the expected volume of inpatient stays in August based on the yearly hospital activity, taking into account the number of calendar days in August. Activity reduction was defined as when the observed volume of stays in August was lower than the 95% confidence interval (95% CI) of the expected volume of stays.

### Statistical analysis

Data manipulation and analyses were performed using SAS software (version 9.2; SAS Institute Inc., Cary, NC). The month was selected as the unit of temporal analysis because it was the information with utmost granularity for marking the time of patient discharge from hospital. The seasonal patterns among the surgical mortality, workers' vacation rate, hospital activity, and maximum temperatures were plotted over time. In interpreting the variation in these metrics, we were expecting to explore potential correlations and determine whether there were any time-dependent effects. Categorical variables were presented using absolute and relative frequencies, and they were compared between August and other months using the χ^2^ test. Continuous variables were presented using the means and standard deviation, and they were compared using the Mann-Whitney test. Estimates were accompanied with the corresponding 95% CI.

To explore the determinants of surgical mortality, we ran multilevel logistic regressions (PROC GLIMMIX), taking into account the clustering effect of patients within hospitals. Death was the outcome of interest in the final model, whereas August was the main predictor, which was entered as either a binary variable (August vs. other months) or categorical variable, depending on the activity reduction at each hospital (August with activity reduction or without activity reduction vs. other months). To control for potential confounders, the patient characteristics (e.g., sex, age, Elixhauser comorbidities, surgical procedure codes, and complexity) were selected a priori as clinically important covariates as well as the year of hospital discharge to consider secular trends and coding variations. Additionally, the outcomes were adjusted for the hospital status and location to account for the case-mix and temperature variations across institutions. Interactions between the main predictor and other covariates entered in the model were tested. The results were presented as adjusted odds ratios (OR) with corresponding 95% CI. Furthermore, a sensitivity analysis was performed on primary outcome measure to account for potential variations in estimates by extending mortality measure periods from 30 to 45 days after patient admission.

## Results

### Characteristics of the patients and hospitals

**Fig 1:**
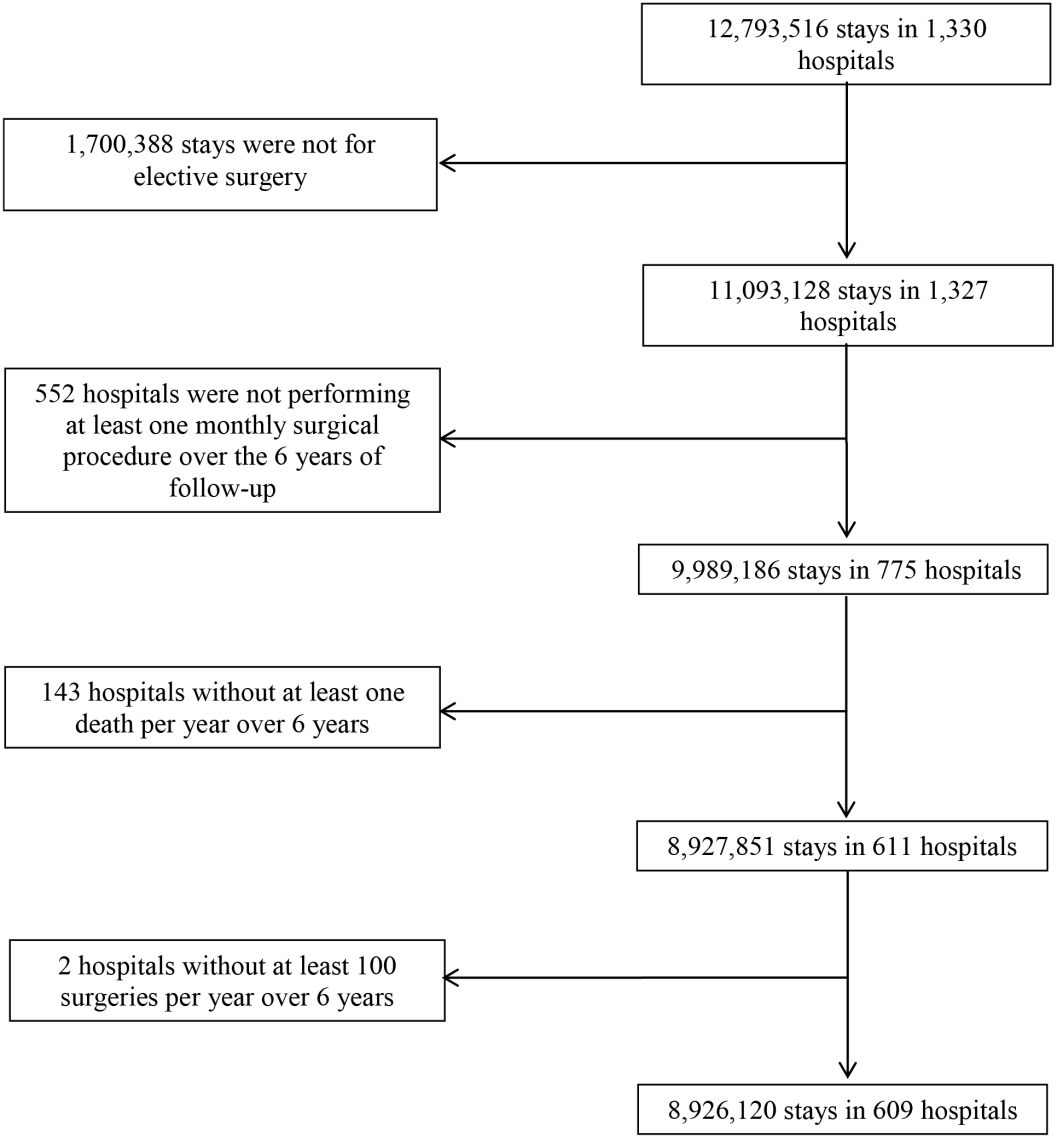
Study flowchart. Flow diagram of hospitals and stays selected for inclusion.

A total of 609 French hospitals had 8,926,120 open elective surgical discharges between 2007 and 2012 (Fig 1). The hospital and inpatient stay characteristics are shown in Table 1. There were 73,477 deaths, accounting for an overall perioperative mortality of 0.8%, ranging from 0.1% to 3.0% per institution. Hospitals demonstrated an average of 237 admissions per month during the study period, and the mean temperature recorded in their respective geographic area was 16.9 degrees Celsius (°C).

**Table 1:** Hospitals and populations studied in France.

**Hospital**	**N = 609**	**%**
Monthly volume of stays per hospital, mean (SD)	237.0 (349.7)	-
Monthly temperature per hospital, mean (SD)	16.9 (1.8)	-
Status		
* Teaching*	48	7.9
* Public or private non-for-profit*	260	42.7
* Private for profit*	301	49.4
Geographic location		
* Paris*	121	19.9
* North West*	102	16.7
* North East*	160	26.3
* South West*	85	14.0
* South East*	141	23.2
**Inpatient stays**	**N = 8,926,120**	**%**
Inpatient mortality	73,477	0.8
Year		
* 2007*	1,545,068	17.3
* 2008*	1,535,327	17.2
* 2009*	1,493,460	16.7
* 2010*	1,459,603	16.4
* 2011*	1,452,632	16.3
* 2012*	1,440,030	16.1
Women	4,648,989	52.1
Age (years), mean (SD)	58.2 (17.7)	-
No. different Elixhauser comorbidities,^a^ mean (SD)	0.8 (1.1)	-
Surgical procedure codes		
* Operation on the Nervous system*	267,287	3.0
* Operation on the Ear*, *Nose*, *Mouth and Pharynx*	481,417	5.4
* Operation on the Cardiovascular system*	1,396,807	15.6
* Operation on the Hematologic and Lymphatic system*	321,201	3.6
* Operation on the Respiratory system*	132,926	1.5
* Operation on the Digestive system*	859,637	9.6
* Operation on the Urinary system*	209,809	2.4
* Operation on the Genital organs*	986,981	11.1
* Operation on the Endocrine system*	274,791	3.1
* Operation on the Musculoskeletal system*	3,494,955	39.2
* Operation on the Integumentary system*	1,326,220	14.9
Procedure complexity, mean (SD)	364.8 (291.4)	-
Month		
* January*	783,138	8.8
* February*	798,006	8.9
* March*	848,363	9.5
* April*	771,396	8.6
* May*	732,360	8.2
* June*	765,809	8.6
* July*	664,904	7.4
* August*	468,758	5.3
* September*	737,604	8.3
* October*	860,629	9.6
* November*	762,169	8.5
* December*	732,984	8.2

^a^ Elixhauser comorbidities include congestive heart failure, cardiac arrhythmias, valvular disease, pulmonary circulation disorders, peripheral vascular disorders, hypertension uncomplicated/complicated, paralysis, other neurological disorders, chronic pulmonary disease, diabetes uncomplicated/complicated, hypothyroidism, renal failure, liver disease, peptic ulcer disease excluding bleeding, AIDS/HIV, lymphoma, metastatic cancer, solid tumour without metastasis, rheumatoid arthritis/collagen vascular diseases, coagulopathy, obesity, weight loss, fluid and electrolyte disorders, blood loss anaemia, deficiency anaemia, alcohol abuse, drug abuse, psychoses, and depression.

### Seasonal patterns

**Fig 2:**
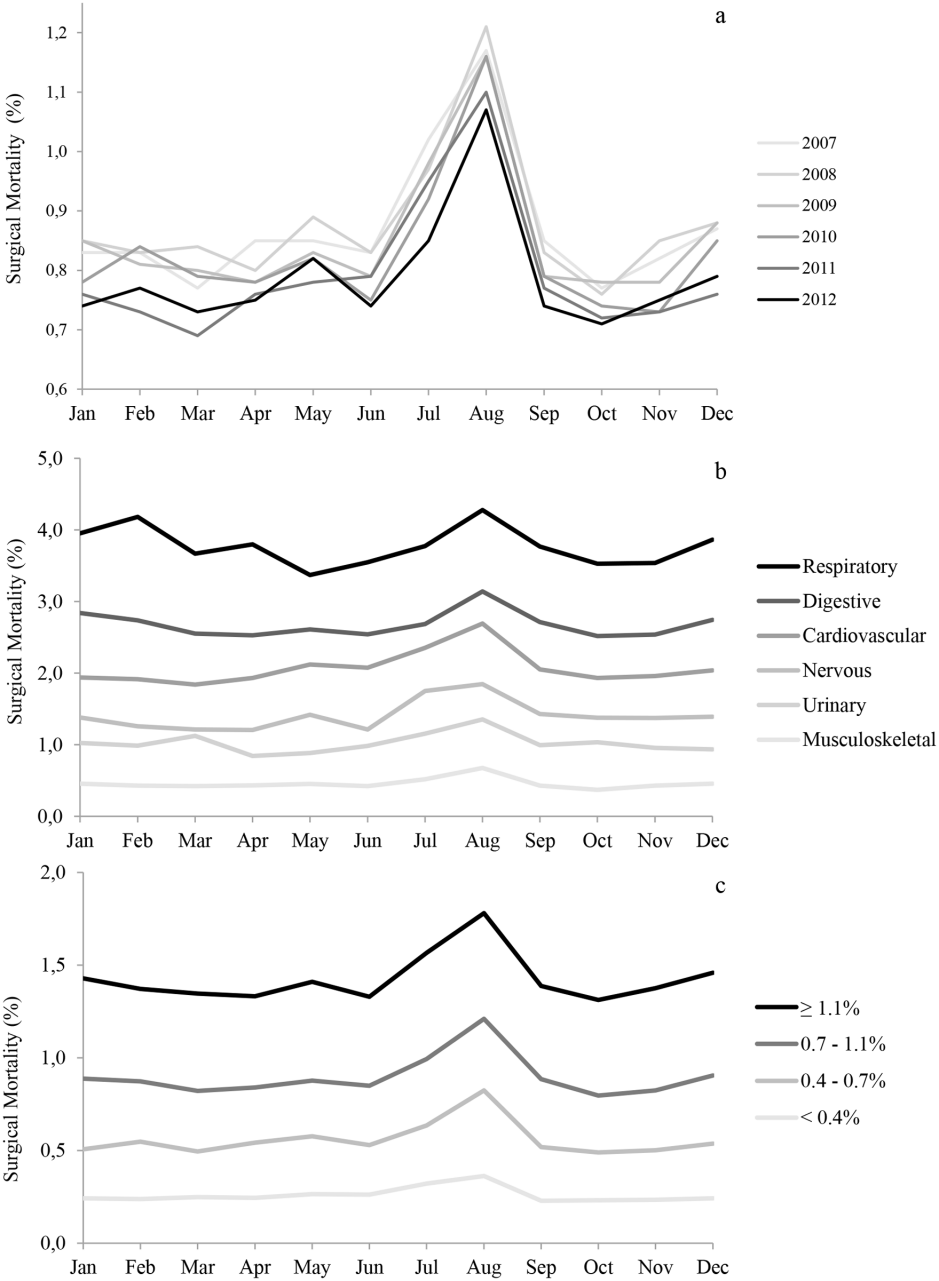
Seasonal pattern of surgical mortality. These figures present the monthly change in crude mortality rate by year (2a), surgical specialty (2b) and quartile of hospital mortality (2c).

Monthly variations in the surgical mortality rate were highly reproducible across years and demonstrated a recurring summer mortality peak (Fig 2a). Across the 6 years studied, the mortality rate in August was 1.15% (95% CI, 1.09–1.20), while it was 0.95% (95% CI, 0.89–1.01, p = .004) in July and 0.81% (95% CI, 0.79–0.82, p<.001) in the other months. Overall, 5,378 deaths occurred in August, representing 1,604 excess deaths (42.5%; 1,604/3,774) relative to what would be expected based on the other months' average rates. Additionally, a mortality peak was observed across the spectrum of various surgical specialties (Fig 2b) and institutions (Fig 2c), whatever the baseline risk of mortality for the patients.

**Fig 3:**
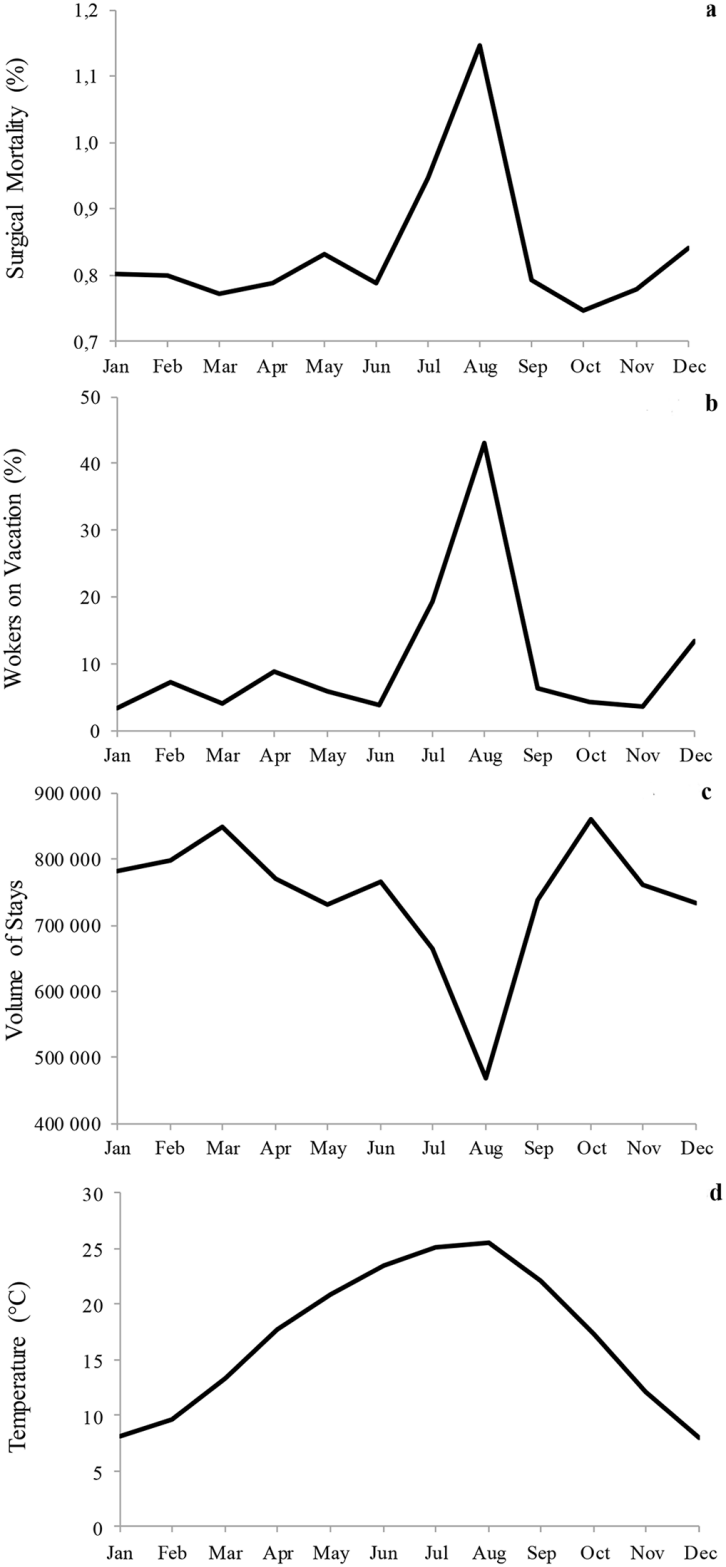
Seasonal pattern of predictors. The figures present the monthly changes in mortality rates (3a), along with those of workers' leave (3b), hospital activity (3c) and maximum temperatures (3d).

We observed the same seasonal patterns regarding workers' leave, mirroring the surgical mortality variations (Fig 3a). The rate of workers on vacation was 43.0% (95% CI, 38.9–47.2) in August, compared with 19.3% (95% CI, 16.3–22.4, p<.001) in July and 7.3% (95% CI, 4.6–10.1, p<.001) in the other months (Fig 3b). In parallel, hospital activity (Fig 3c) decreased substantially in August (78,126 inpatient stays in average; 95%CI, 75,298–80,954) compared to July (110,817; 95% CI, 106,727–114,907, p = .004) and the other months (128,142; 95CI% 125,697–130,586, p<.001). With respect to temperature (Fig 3d), the warmer months were August (25.6°C; CI 95%, 25.4–25.9) and July (25.2°C; 95%CI, 24.9–25.4) compared with the other months (16.1°C; 15.9–16.2, p<.001).

### “August effect”

Among all patients hospitalised for elective surgery, 468,758 (5.3%) were admitted in August, including 422,117 (90.1%) stays in hospitals with activity reduction and 46,641 (9.9%) stays in hospitals without activity reduction. There were fewer women and musculoskeletal procedures in August, while digestive and integumentary surgeries were more frequent (Table 2). Furthermore, the market share for private hospitals was reduced in August relative to that of teaching and public institutions.

**Table 2:** Population comparison between August and other months.

Characteristics^a^	Other Months Mean N = 768,851 (%)	August N = 468,758 (%)
Hospital geographic location		
* Paris*	119,292 (15.5)	62,736 (13.4)
* North west*	158,161 (20.6)	98,808 (21.1)
* North east*	170,139 (22.1)	112,016 (23.9)
* South west*	122,022 (15.9)	77,426 (16.5)
* South east*	199,238 (25.9)	117,772 (25.1)
Hospital status		
* Teaching*	193,743 (25.2)	135,150 (28.8)
* Public or private non-for-profit*	188,714 (24.5)	120,686 (25.7)
* Private for profit*	386,394 (50.3)	212,922 (45.4)
Year		
* 2007*	132,938 (17.3)	82,754 (17.7)
* 2008*	132,344 (17.2)	79,542 (17.0)
* 2009*	128,653 (16.7)	78,279 (16.7)
* 2010*	125,765 (16.4)	76,188 (16.3)
* 2011*	125,135 (16.3)	76,143 (16.2)
* 2012*	124,016 (16.1)	75,852 (16.2)
Women	401,291 (52.2)	234,785 (50.1)
Age (years), mean (SD)	58.2 (17.7)	58.2 (18.6)
No. different Elixhauser comorbidities,^b^ mean (SD)	0.8 (1.1)	0.8 (1.2)
Surgical procedure codes		
* Operation on the nervous system*	22,885 (3.0)	15,550 (3.3)
* Operation on the ear*, *nose*, *mouth and pharynx*	41,667 (5.4)	23,078 (4.9)
* Operation on the cardiovascular system*	120,218 (15.6)	74,414 (15.9)
* Operation on the hematologic and lymphatic system*	27,260 (3.5)	21,341 (4.6)
* Operation on the respiratory system*	11,232 (1.5)	9,373 (2.0)
* Operation on the digestive system*	73,106 (9.5)	55,466 (11.8)
* Operation on the urinary system*	18,047 (2.3)	11,290 (2.4)
* Operation on the genital organs*	85,180 (11.1)	50,006 (10.7)
* Operation on the endocrine system*	23,910 (3.1)	11,786 (2.5)
* Operation on the musculoskeletal system*	302,326 (39.3)	169,365 (36.1)
* Operation on the integumentary system*	113,465 (14.8)	78,110 (16.7)
Procedure complexity, mean (SD)	365.1 (290.8)	359.8 (301.8)

^a^ p<.001 for all variables.

^b^ Elixhauser comorbidities include congestive heart failure, cardiac arrhythmias, valvular disease, pulmonary circulation disorders, peripheral vascular disorders, hypertension uncomplicated/complicated, paralysis, other neurological disorders, chronic pulmonary disease, diabetes uncomplicated/complicated, hypothyroidism, renal failure, liver disease, peptic ulcer disease excluding bleeding, AIDS/HIV, lymphoma, metastatic cancer, solid tumour without metastasis, rheumatoid arthritis/collagen vascular diseases, coagulopathy, obesity, weight loss, fluid and electrolyte disorders, blood loss anaemia, deficiency anaemia, alcohol abuse, drug abuse, psychoses, and depression.

Based on a multivariate analysis, there was a reduction in the risk of postoperative death from 2007 through 2012 (Table 3). Patient characteristics that were independently associated with death included male gender, increasing age, Elixhauser comorbidities, and surgical procedure complexity. The riskier procedures were operations on the respiratory, digestive, nervous, or cardiovascular systems. After adjusting for all covariates, we found an “August effect”, reflecting a higher risk for patients undergoing operations at this time (OR 1.16; 95% CI, 1.12–1.19, p<.001). Additionally, hospital activity reduction in August was associated with increased mortality (OR ranging from 1.15 to 1.36, p<.001), while death risk in hospitals without activity reduction was not significant (OR 1.06; 95% CI, 0.97–1.16). On the other hand, the hospital geographic location did not influence the occurrence of inpatient death in August compared with other months. We found interactions between the variables entered in the model related to sex, comorbidities, age and operations on the hematologic, lymphatic, cardiovascular, digestive, and musculoskeletal system. The “August effect” persisted in all corresponding strata and by extending the mortality period to 45 days after patient admission (see S1 Fig and S1 Table).

**Table 3:** Factors independently associated in-hospital death within 30 days.

Characteristics^a^	OR (95% CI)
August (Ref = Other months)	1.16 (1.12–1.19)
*Hospitals with activity reduction of 20–39% in August*	1.15 (1.10–1.20)
*Hospitals with activity reduction of 40–59% in August*	1.18 (1.12–1.24)
*Hospitals with activity reduction of 60% in August*	1.36 (1.16–1.58)
Hospital geographic location (Ref = Paris)	
*North west*	0.89 (0.77–1.02)
*North east*	0.89 (0.78–1.01)
*South west*	0.88 (0.76–1.01)
*South east*	1.02 (0.90–1.16)
Hospital status (Ref = Private for profit)	
*Teaching*	1.67 (1.44–1.93)
*Public or Private non-for-profit*	1.82 (1.68–1.97)
Year of hospital discharge (Ref = 2012)	
*2007*	1.46 (1.42–1.50)
*2008*	1.39 (1.35–1.43)
*2009*	1.24 (1.21–1.28)
*2010*	1.16 (1.13–1.19)
*2011*	1.09 (1.06–1.12)
Women (Ref = Men)	0.80 (0.79–0.81)
Age (by 1 year increase)	1.06 (1.05–1.06)
No. different Elixhauser comorbidities (by 1 comorbidity increase)^b^	1.59 (1.59–1.60)
Surgical procedure codes (Ref = Other site)	
*Operation on the nervous system*	4.02 (3.87–4.18)
*Operation on the ear*, *nose*, *mouth and pharynx*	0.82 (0.77–0.88)
*Operation on the cardiovascular system*	2.53 (2.47–2.60)
*Operation on the hematologic and lymphatic system*	0.80 (0.76–0.83)
*Operation on the respiratory system*	5.61 (5.41–5.82)
*Operation on the digestive system*	5.33 (5.21–5.46)
*Operation on the urinary system*	1.42 (1.35–1.49)
*Operation on the genital organs*	0.45 (0.43–0.47)
*Operation on the endocrine system*	0.38 (0.33–0.43)
*Operation on the musculoskeletal system*	1.05 (1.02–1.07)
*Operation on the integumentary system*	1.21 (1.17–1.25)
Procedure complexity (by 100 unit increase)	1.04 (1.04–1.04)

^a^ p<.001 for all variables excepted hospital geographic location, based on multilevel logistic regression model.

^b^ Elixhauser comorbidities include congestive heart failure, cardiac arrhythmias, valvular disease, pulmonary circulation disorders, peripheral vascular disorders, hypertension uncomplicated/complicated, paralysis, other neurological disorders, chronic pulmonary disease, diabetes uncomplicated/complicated, hypothyroidism, renal failure, liver disease, peptic ulcer disease excluding bleeding, AIDS/HIV, lymphoma, metastatic cancer, solid tumour without metastasis, rheumatoid arthritis/collagen vascular diseases, coagulopathy, obesity, weight loss, fluid and electrolyte disorders, blood loss anaemia, deficiency anaemia, alcohol abuse, drug abuse, psychoses, and depression.

## Discussion

We found an increased risk of death for patients in France who undergo elective surgery during August, evidenced through a recurring annual peak in mortality across the main surgical specialties. This pattern would result from two primary factors, after simultaneously adjusting the outcomes for the patient, procedure and institution characteristics.

First, temporary understaffing due to massive holiday departure can cause an excess in hospital mortality if it is not properly managed. Since 1969, French workers have been allowed to take four weeks of paid leave per year. Currently, they are entitled to 30 vacation days annually, which is more than in most European countries [8]. What is remarkable is the concentration of their leave and the potential issue that traditionally a majority of the French population takes a full month of vacation during summer, particularly in August [9]. As a consequence, inadequate matching of hospital staff with patient needs can jeopardise care safety [11] and overwhelm the remaining health personnel [19]. Although recruitment of interim staff represents a flexible alternative for tackling momentary staff reduction, this solution is expensive and can disrupt regular team dynamics as interim healthcare workers are unfamiliar with a particular hospital's context. Especially, an unexpectedly high turnover rate of operating room members during this period may impact the surgical outcomes [20]. Compared with other countries, this does not necessarily reflect trainees rotating or newly qualified doctors moving to new posts, which normally occurs in November in the French institutions [21]. However, it appears plausible that the most experienced surgeons are more likely to take their summer leave in August than their youngest colleagues, leading to suboptimal care at this period.

Second, we found that patients who underwent surgery in hospitals with significant activity reduction in August presented with higher mortality. The more the hospital volume of procedures decreased in August, the more the patients were at risk of dying. A potential explanation is that those institutions unable to maintain a regular volume of surgical procedures over the year would not provide safe care at any time. Slowing down activity below a critical threshold [12], in conjunction with bed closure and temporary staff reduction during summer vacation, may disorganise the clinical pathways. In turn, this may induce life-threatening situations and impair the capacity of surgical teams or intensive care units for appropriately managing harmed patients [22].

Although our analysis considered climate factors, similar patterns in seasonal mortality were evidenced across the years, and the repetition of the “August effect” was not influenced by anomalous events [3] such as occasional heat waves. In addition, we observed an increased mortality in August relative to July, while there were equivalent temperatures over these months. Similarly, there was no effect related to the hospitals’ geographic location based on multivariate modelling in spite of the well-established temperature gradient from the north to south of France during the summer. Therefore, we consider that the hospital mortality increase was not related to high temperatures in August [23], which seems consistent for elective surgery performed in an air-conditioned environment.

In conducting a large population-based study and observing the repetition of significant seasonal patterns over the years, we acknowledge several limitations to our study. We were not able to control for provider characteristics that impact the outcomes, such as the team composition or surgeon’s experience [20, 24]. Accurate data on the surgical staff available at the time of surgery would be a step to highlighting the direct link between personnel staffing and patient mortality at the hospital level. Furthermore, we monitored the in-hospital mortality over a broad range of procedures without knowing the prognosis of patients outside the hospital or separating preventable from inevitable deaths [25]. This point is critical, as we assumed that hospital-wide mortality was a valid metric of patient safety [26]. Risk adjustment can only account for factors that can be identified and measured accurately [27]. Although we considered the patient characteristics and procedural complexity in the adjustment scheme, this may not be sufficient to ascertain the impact of the differential case mix on the surgical outcome. “Do not resuscitate” designations and detailed information about the presence of diagnoses on admission were not available in the administrative data for adjusting mortality [28]. Emergency admissions were excluded from our initial dataset, but there was no means for considering quasi-emergency cases, namely high-risk patients requiring early surgical treatment. Accordingly, reductions in the hospital procedural volumes during summer may reflect the postponement of true elective cases until after the holidays; as a result, there is a preponderance of semi-urgent cases with a poorer prognosis performed in the August timeframe. However, inpatient mortality was adjusted for several risk factors (e.g., cancer) and we observed the same seasonal pattern across a broad range of surgical specialties and hospitals.

Despite observing a reduction in the risk of postoperative death over the years [29], the recurring August mortality peak in France raises important questions about how to maintain hospital activity and optimal staffing levels. Mandatory measures have been implemented to deal with hazardous situations. Although these threats to population health have a very low probability of occurrence, every hospital has to develop plans for managing crisis situations, such as epidemic outbreaks, natural disasters, terrorism attacks or other major accidents [30]. Conversely, poor awareness of common safety issues that can be spotted by repeated patterns over time may be associated with a lack of interest from health authorities. Such seasonal disruptions in care process are predictable every year and could be regulated through better coordination of human activities. Reductions in the volume of elective surgery may result from vacation leaves among patients and healthcare workers. Both hospital staff and patients prefer to have care coordinated outside of the August summer vacation time period. Rather than proposing the unpopular solution of suspending annual leaves in August, a fairly straightforward way of eradicating this problem would consist of balancing vacations over the course of the year. On the condition of overcoming both cultural and political barriers, resolving the “August effect” will require a smoothing of the volume of surgical procedures over months and a more rational scheduling of worker vacations.

## Supporting Information

S1 Fig Sensitivity analysis on primary outcome. Seasonal pattern of surgical mortality at 30 days and 45 days.
(TIF)

S1 Table Sensitivity analysis on primary outcome. Factors independently associated with in-hospital death within 30 days and 45 days after patient admission.
(DOCX)
