# Supplementary figures and images for: Increased Mortality for Elective Surgery during Summer Vacation: A Longitudinal Analysis of Nationwide Data
Source: PLoS One. 2015 Sep 25;10(9):e0137754. doi: 10.1371/journal.pone.0137754 (PMC4583258; doi:10.1371/journal.pone.0137754)

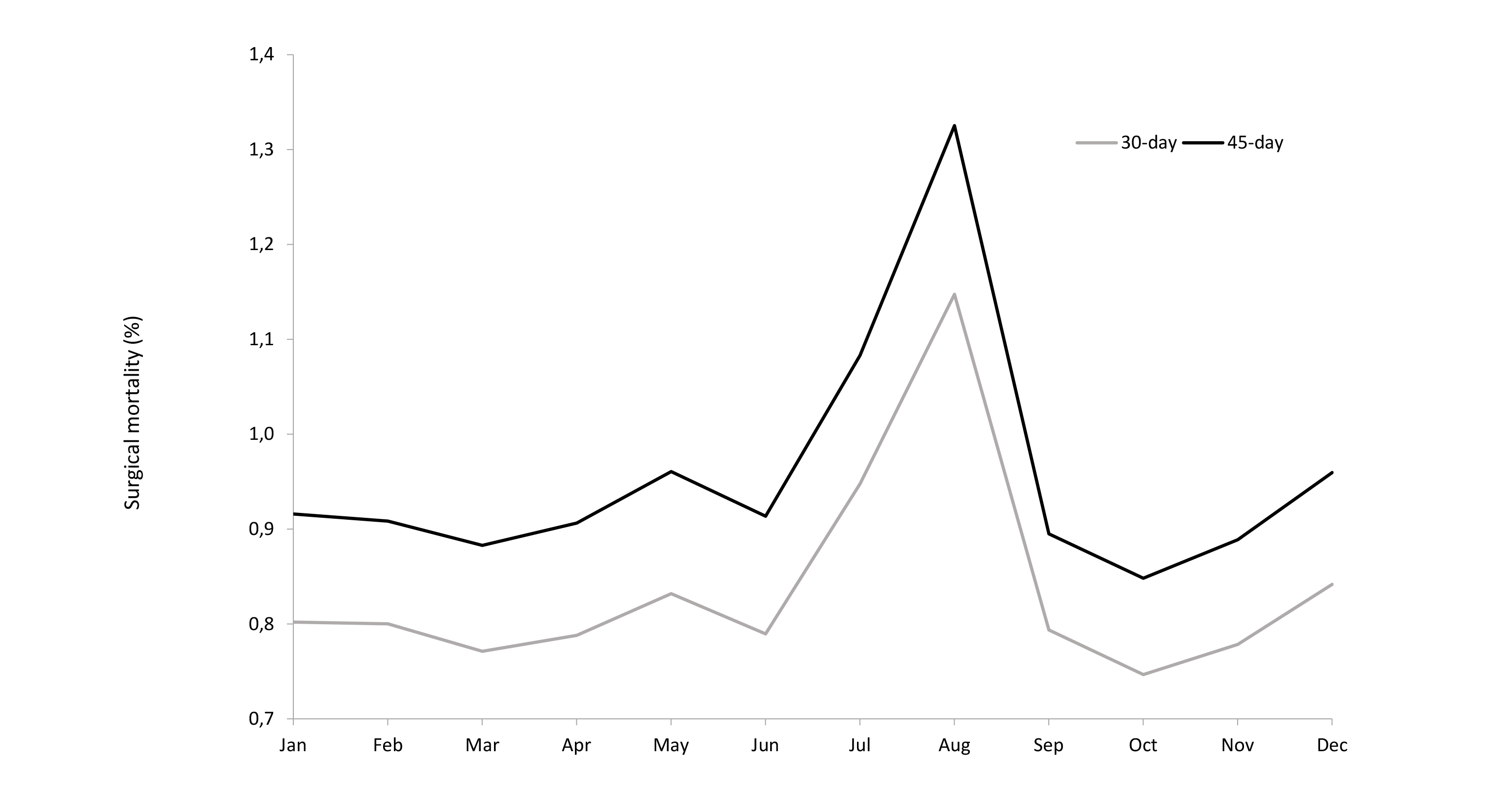

Supplement: S1 Fig — Seasonal pattern of surgical mortality at 30 days and 45 days. (TIF) [file pone.0137754.s001.tif]
