# Supplementary material for: Increased Mortality for Elective Surgery during Summer Vacation: A Longitudinal Analysis of Nationwide Data
Source: PLoS One. 2015 Sep 25;10(9):e0137754. doi: 10.1371/journal.pone.0137754 (PMC4583258; doi:10.1371/journal.pone.0137754)
Supplement: S1 Table — Factors independently associated with in-hospital death within 30 days and 45 days after patient admission. (DOCX) [file pone.0137754.s002.docx]

**S1 Table. Sensitivity analysis on primary outcome.** Factors independently associated with in-hospital death within 30 days and 45 days after patient admission.

|  | **30 Days** | **45 days** |
| --- | --- | --- |
| **Characteristics**^a^ | **OR (95% CI)** | **OR (95% CI)** |
| August (Ref=Other months) | 1.16 (1.12 - 1.19) | 1.16 (1.13 - 1.19) |
| *Hospitals with activity reduction of 20 -39% in August* | 1.15 (1.10 - 1.20) | 1.15 (1.11 - 1.19) |
| *Hospitals with activity reduction of 40 - 59% in August* | 1.18 (1.12 - 1.24) | 1.18 (1.12 - 1.24) |
| *Hospitals with activity reduction of 60% in August* | 1.36 (1.16 - 1.58) | 1.46 (1.26 - 1.68) |
| Hospital geographic location (Ref=Paris) |  |  |
| *North west* | 0.89 (0.77 - 1.02) | 0.88 (0.77 - 1.00) |
| *North east* | 0.89 (0.78 - 1.01) | 0.88 (0.76 - 1.01) |
| *South west* | 0.88 (0.76 - 1.01) | 0.89 (0.78 - 1.01) |
| *South east* | 1.02 (0.90 - 1.16) | 1.01 (0.89 - 1.15) |
| Hospital status (Ref=Private for profit) |  |  |
| *Teaching* | 1.67 (1.44 - 1.93) | 1.68 (1.46 - 1.94) |
| *Public or Private non-for-profit* | 1.82 (1.68 - 1.97) | 1.86 (1.71 - 2.01) |
| Year of hospital discharge (Ref=2012) |  |  |
| *2007* | 1.46 (1.42 - 1.50) | 1.44 (1.41 - 1.48) |
| *2008* | 1.39 (1.35 - 1.43) | 1.37 (1.34 - 1.40) |
| *2009* | 1.24 (1.21 - 1.28) | 1.24 (1.21 - 1.27) |
| *2010* | 1.16 (1.13 - 1.19) | 1.16 (1.13 - 1.19) |
| *2011* | 1.09 (1.06 - 1.12) | 1.09 (1.06 - 1.12) |
| Women (Ref=Men) | 0.80 (0.79 - 0.81) | 0.80 (0.79 - 0.81) |
| Age (by 1 year increase) | 1.06 (1.05 - 1.06) | 1.05 (1.05 - 1.05) |
| No. different Elixhauser comorbidities (by 1 comorbidity increase)^b^ | 1.59 (1.59 - 1.60) | 1.59 (1.59 - 1.60) |
| Surgical procedure codes (Ref=Other site) |  |  |
| *Operation on the nervous system* | 4.02 (3.87 - 4.18) | 3.82 (3.68 - 3.96) |
| *Operation on the ear, nose, mouth and pharynx* | 0.82 (0.77 - 0.88) | 0.74 (0.70 - 0.79) |
| *Operation on the cardiovascular system* | 2.53 (2.47 - 2.60) | 2.54 (2.49 - 2.60) |
| *Operation on the hematologic and lymphatic system* | 0.80 (0.76 - 0.83) | 0.79 (0.75 - 0.82) |
| *Operation on the respiratory system* | 5.61 (5.41 - 5.82) | 6.01 (5.81 - 6.21) |
| *Operation on the digestive system* | 5.33 (5.21 - 5.46) | 5.07 (4.97 - 5.19) |
| *Operation on the urinary system* | 1.42 (1.35 - 1.49) | 1.42 (1.36 - 1.48) |
| *Operation on the genital organs* | 0.45 (0.43 - 0.47) | 0.45 (0.43 - 0.47) |
| *Operation on the endocrine system* | 0.38 (0.33 - 0.43) | 0.39 (0.35 - 0.43) |
| *Operation on the musculoskeletal system* | 1.05 (1.02 - 1.07) | 1.03 (1.01 - 1.06) |
| *Operation on the integumentary system* | 1.21 (1.17 - 1.25) | 1.20 (1.17 - 1.24) |
| Procedure complexity (by 100 unit increase) | 1.04 (1.04 - 1.04) | 1.03 (1.03 - 1.03) |

^a^ p<.001 for all variables excepted hospital geographic location, based on multilevel logistic regression model.

^b^ Elixhauser comorbidities include congestive heart failure, cardiac arrhythmias, valvular disease, pulmonary circulation disorders, peripheral vascular disorders, hypertension uncomplicated/complicated, paralysis, other neurological disorders, chronic pulmonary disease, diabetes uncomplicated/complicated, hypothyroidism, renal failure, liver disease, peptic ulcer disease excluding bleeding, AIDS/HIV, lymphoma, metastatic cancer, solid tumour without metastasis, rheumatoid arthritis/collagen vascular diseases, coagulopathy, obesity, weight loss, fluid and electrolyte disorders, blood loss anaemia, deficiency anaemia, alcohol abuse, drug abuse, psychoses, and depression.
